# Disordered directional brain network interactions during learning dynamics in schizophrenia revealed by multivariate autoregressive models

**DOI:** 10.1002/hbm.25032

**Published:** 2020-05-21

**Authors:** Shahira J. Baajour, Asadur Chowdury, Patricia Thomas, Usha Rajan, Dalal Khatib, Caroline Zajac‐Benitez, Dimitri Falco, Luay Haddad, Alireza Amirsadri, Steven Bressler, Jeffery A. Stanley, Vaibhav A. Diwadkar

**Affiliations:** ^1^ Department of Psychiatry and Behavioral Neuroscience Wayne State University School of Medicine Detroit Michigan USA; ^2^ Center for Complex Systems and Brain Sciences Florida Atlantic University Boca Raton Florida USA; ^3^ Department of Psychology Florida Atlantic University Boca Raton Florida USA

**Keywords:** associative learning, cortical‐hippocampal networks, fMRI, learning dynamics, multivariate autoregressive models, schizophrenia

## Abstract

Directional network interactions underpin normative brain function in key domains including associative learning. Schizophrenia (SCZ) is characterized by altered learning dynamics, yet dysfunctional directional functional connectivity (dFC) evoked during learning is rarely assessed. Here, nonlinear learning dynamics were induced using a paradigm alternating between conditions (Encoding and Retrieval). Evoked fMRI time series data were modeled using multivariate autoregressive (MVAR) models, to discover dysfunctional direction interactions between brain network constituents during learning stages (Early vs. Late), and conditions. A functionally derived subnetwork of coactivated (healthy controls [HC] ∩ SCZ] nodes was identified. MVAR models quantified directional interactions between pairs of nodes, and coefficients were evaluated for intergroup differences (HC ≠ SCZ). In exploratory analyses, we quantified statistical effects of neuroleptic dosage on performance and MVAR measures. During Early Encoding, SCZ showed reduced dFC within a frontal–hippocampal–fusiform network, though during Late Encoding reduced dFC was associated with pathways *toward* the dorsolateral prefrontal cortex (dlPFC). During Early Retrieval, SCZ showed increased dFC in pathways to and from the dorsal anterior cingulate cortex, though during Late Retrieval, patients showed increased dFC in pathways *toward* the dlPFC, but decreased dFC in pathways *from* the dlPFC. These discoveries constitute novel extensions of our understanding of task‐evoked dysconnection in schizophrenia and motivate understanding of the *directional* aspect of the dysconnection in schizophrenia. Disordered directionality should be investigated using computational psychiatric approaches that complement the MVAR method used in our work.

## Graphical abstract

Coactivation patterns based on a conjunction analyses (left) reveal highly significant overlap between schizophrenia patients and healthy controls. However, when within the network formed by coactivated clusters (right), we investigated directional functional connectivity, substantive differences emerge.

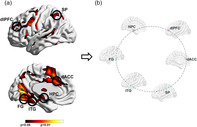

## 1 INTRODUCTION

Schizophrenia (SCZ) (Saha, Chant, Welham, & McGrath, 2005; Schultz & Andreasen, 1999) is characterized by prominent deficits in cognitive domains including learning and memory (Aleman, Hijman, de Haan, & Kahn, 1999; Brambilla et al., 2011), that are central to its core (Ragland et al., 2012). These deficits are associated with dysfunction of brain regions including the hippocampus, the dorsolateral prefrontal cortex (dlPFC), and the dorsal anterior cingulate cortex (dACC) (Diwadkar et al., 2008; Heckers et al., 1998; Konradi & Heckers, 2003; Ragland et al., 2009; Ragland et al., 2017; Woodcock, Wadehra, & Diwadkar, 2016), and interactions between them. The cumulative effects are consistent with the “dysconnection” hypothesis (Friston, Brown, Siemerkus, & Stephan, 2016; Robison, Thakkar, & Diwadkar, 2019; Rolls et al., 2019). By inference, deficits in learning and memory are related to dysfunction in the integrative tone of selected networks, or the inability of reentrant functional connections to interact within the system (Érdi, Ujfalussy, & Diwadkar, 2009).

Functional network transactions are fundamentally *directional* in nature (Friston, 2011; Park & Friston, 2013). Yet, a preponderance of studies investigating task‐related dysconnection during learning and memory rely on undirected functional connectivity (uFC) (Silverstein, Bressler, & Diwadkar, 2016; Wadehra, Pruitt, Murphy, & Diwadkar, 2013). uFC seeks to capture statistical relationships between fluctuations in BOLD activity between different brain areas, and is typically represented by zero‐lag bivariate correlations between pairs of regions (A, B). uFC models are agnostic with respect to any directionality of effects (A ➔ B vs. B ➔ A). Several studies have investigated statistical relationships between resting‐state FC (Samudra et al., 2015) and psychological performance on tasks of relational memory (Avery, Rogers, & Heckers, 2018). The resultant discoveries from fMRI signals have been largely agnostic regarding plausible directional interactions between constituents of learning related networks. Analyses of brain‐wide resting‐state fMRI data suggest that the dysconnectome in SCZ, is pervasive and global (Ji et al., 2019; Rolls et al., 2019). These compelling results motivate the search for specific task‐induced dysfunction, because resting‐state connectomics do not predict task‐evoked dysfunction in “linear” ways (Hermundstad et al., 2013; Park & Friston, 2013); indeed, in SCZ, changes in uFC during task‐based processing are not straightforwardly predicted by differences in resting‐state FC in the same patients (Salomon et al., 2011).

To address this lacuna, here fMRI time series data acquired during an associative learning paradigm were submitted to directional functional connectivity (dFC) analyses based on the application of multivariate autoregressive (MVAR) models (Asemi, Ramaseshan, Burgess, Diwadkar, & Bressler, 2015; Diwadkar, Asemi, Burgess, Chowdury, & Bressler, 2017). These models are well suited for assessing such interactions between any nodes in any class of network with quantifiable dynamics (Bressler & Seth, 2011). The employed task was notable for its reliance on *both* relational memory (Avery et al., 2019) *and* the resultant nonlinear learning dynamics induced by having to *learn* associations over time (Stephan, Baldeweg, & Friston, 2006).

Associative learning is driven by the long‐term potentiation (LTP) of synaptic strengths, modified in regions including the hippocampus, the prefrontal cortex and subcortical structures including the basal ganglia (Gruart, Leal‐Campanario, Lopez‐Ramos, & Delgado‐Garcia, 2015; Izquierdo & Medina, 1997), and is controlled by the excitatory role of the *N*‐methyl‐d‐aspartate (NMDA) receptor which drives LTP (Silva, 2003). How the molecular mechanisms of learning (primarily derived from rodent models) cascade “upward” to the mesoscopic and macroscopic scales is unclear (Singh, 2012). Nevertheless, fMRI studies and pharmacologic challenges (using ketamine, an NMDA receptor antagonist) repeatedly (a) confirm the role of frontal and hippocampal regions in learning (Woodcock, White, & Diwadkar, 2015) and learning dynamics (Banyai, Diwadkar, & Érdi, 2011), and (b) the role of NMDA in sub serving learning proficiency (Krystal et al., 1994; Krystal et al., 1999).

As has been lucidly noted in many discussions on the neuroscience of brain networks (Park & Friston, 2013; Singh, 2012), any class of “neural” activity relating to any domain unfolds at multiple spatial, temporal, and mechanistic scales. In no domain is this aspect truer than in the study of the molecular, neurochemical and computational bases of learning and memory (Banyai et al., 2011; Chen & Tonegawa, 1997; Diwadkar et al., 2008; Ranganath, Minzenberg, & Ragland, 2008; Silva, 2003). Notably, both SCZ *and* deficits in learning and memory are associated with NMDA receptor hypofunction (Brambilla, Riva, Melcangi, & Diwadkar, 2007; Harrison, Law, & Eastwood, 2003; Stephan et al., 2006). More fundamentally, in SCZ, glutamatergic dysfunction may be a pathological bridge between core clinical symptomatology and behavioral deficits (Limongi et al., In press). Indeed, the glutamate, along with the dopaminergic hypothesis (Howes & Kapur, 2009) represents one of the core theories of the molecular pathophysiology of SCZ (Coyle, 1996), and suggests that the full expression of illness dysfunction is at once, neurochemical (molecular), network (macroscopic), and “computational” (or behavioral). The last level is most proximate to the manifestation of the illness because psychosis is proposed to result from a decreased precision in the encoding of prior beliefs relative to the sensory data, thereby driving maladaptive inferences or “prediction errors” (Friston, Stephan, Montague, & Dolan, 2014; Sterzer et al., 2018). The resultant effects on perceptual, decision and sensorimotor domains are widely documented (Limongi, Bohaterewicz, Nowicka, Plewka, & Friston, 2018; Thakkar, Diwadkar, & Rolfs, 2017), but may generalize to higher level cognitive domains such as learning that frequently rely on frontal–striatal–hippocampal interactions, and are “downstream” from perceptual processing (Heinz et al., 2019). In this vein, SCZ is notably both a neuropsychiatric condition *and* a “model” of pathological brain network interactions (Silverstein et al., 2016; Stephan et al., 2016).

Here, we used an established associative learning paradigm (Diwadkar et al., 2016) to induce classic negatively accelerated learning (Buchel, Coull, & Friston, 1999) characterized by rapid rates of improvements in trial‐on‐trial performance during initial phases, but diminished rates during later phases. These nonlinear behavioral dynamics are notable for distinguishing between early (linear regime) and later stages of learning (an asymptotic regime) (Ravishankar et al., 2019; Stanley et al., 2017). To avoid activation‐related biases from confounding intergroup differences in connectivity, a functionally derived network was employed to identify common activated loci across groups (HC ∩ SCZ) and task conditions (Morris et al., 2018). From this network, times series' were submitted to analyses using MVAR models (Bressler, Richter, Chen, & Ding, 2007; Tang, Bressler, Sylvester, Shulman, & Corbetta, 2012). MVAR models (analogous to Granger causality) rely on principles of temporal precedence in time series data to estimate “causality” between system constituents (we use the weaker term “directionality” in referring to these effects) (Deshpande & Hu, 2012; Roebroeck, Formisano, & Goebel, 2005). Our analyses separately estimate dFC relating to memory Encoding and Retrieval, (see Section 2) and the previously motivated Early and Late phases of learning.

## 2 METHODS AND MATERIALS

### 2.1 Participants

Wayne State University's IRB approved all procedures. Participants (*N* = 55) provided informed consent and were compensated for their participation. HC participants were (by definition) free of psychiatric or neurological conditions (*n* = 24; mean age: 28 years; range: 18–45; nine females; mean full‐scale IQ [FSIQ]: 101.29 [±10.55]; mean PANSS composite score: −0.09 [±1.04]; mean PANSS general score: 16.74 [±1.79]; mean PANSS negative score: 7.74 [±0.86]; mean PANSS positive score: 7.65 [±1.03]). SCZ patients were identified by the treating physicians (A. A. and L. H.) and the diagnosis was confirmed by a research psychologist (U. R.) using the DSM‐V criteria for SCZ (American Psychiatric Association, 2013) (SCZ; *n* = 31; mean age: 29 years; range: 18–50; 10 females; mean FSIQ: 87.74 [±6.06]; mean PANSS composite score: 0.19 [±3.81]; mean PANSS general score: 23.52 [±4.95]; mean PANSS negative score: 12.65 [±3.52]; mean PANSS positive score: 12.84 [±3.13]). All patients were stabilized on a regimen of atypical antipsychotics (Risperidone, Olanzapine, or Aripiprazole). Groups did not differ in age (*p* > .10, see Table 1).

**TABLE 1:** The demographic characteristics for each group are shown. We also show the medication profiles for SCZ patients. All patients (*n* = 31) were stabilized on a regimen of atypical antipsychotics at the time of data acquisition. HC were free of all medications except for antihistamines (*n* = 1)

	SCZ (*n* = 31)	HC (*n* = 24)
*Demographics*		
Age (years)	29.36 (±7.99)	27.72 (±6.33)
Sex (% female)	10 (32%)	9 (38%)
IQ	84.74 (±6.06)	101.29 (±10.55)
*Medication*		
Medicated (%)	31 (100%)	
Antidepressant	6 (19%)	
Antipsychotic	31 (100%)	
Anxiolytic	7 (23%)	
Mood stabilizer	7 (23%)	
CNS stimulant	1 (3%)	
Antihistamines	3 (10%)	
Hypnotics and sedatives	3 (10%)	
Anticholinergic	3 (10%)	
Antihypertensives	3 (10%)	

Abbreviations: HC, healthy controls; SCZ, schizophrenia.

### 2.2 MRI acquisition

Data (3 T Siemens Verio scanner, 32‐channel volume head coil) were acquired using a multiband gradient EPI sequence (TR = 3 s, TE = 24.6 s, multiband factor = 3, FOV = 192 × 192 mm^2^, matrix = 96 × 96, 64 axial slices, resolution = 2 mm^3^). T_1_‐weighted MRI images were collected for normalization and coregistration with the EPI scan (3D Magnetization Prepared Rapid Gradient Echo sequence, TR = 2,150 ms, TE = 3.5 ms, TI = 1,100 ms, flip angle = 8°, FOV = 256 × 256 × 160 mm^3^, 160 axial slices, resolution = 1 mm^3^).

### 2.3 Data processing

Image processing was undertaken in SPM 12 using established methods for temporal (slice timing correction) and spatial preprocessing. EPI images were manually oriented to the AC‐PC line with the reorientation vector applied across the EPI image set, realigned to a reference image to correct for head movement, and coregistered to the anatomical high‐resolution T_1_ image. The T_1_ image was normalized to the MNI template, with the resultant deformations applied to the coregistered EPI images. Low frequency components were removed (low‐pass filter: 128 s) and images were smoothed using a Gaussian filter (8 mm full‐width half maximum). An autoregressive AR(1) model was used to account for serial correlation.

### 2.4 Associative learning

Network dynamics were induced using an object‐location associative learning paradigm (Ravishankar et al., 2019; Stanley et al., 2017; Wadehra et al., 2013; Woodcock et al., 2015), alternating between Encoding, Rest, and Retrieval epochs (27 s each). During encoding epochs, nine objects were presented in their associated locations for naming (3 s/object). Following a brief instruction‐free retention interval (27 s), retrieval was induced by randomly cuing locations and requiring participants to name the associated object. Following another instruction‐free rest interval (27 s), the cycle of epochs was repeated. Eight cycles were used to promote asymptotic performance. The paradigm strongly elicits frontal–hippocampal mechanisms of memory formation, consolidation, and recall (Simons & Spiers, 2003) and is characterized by negatively accelerated learning, which in turn permits the assessment of task‐related dynamics that may differ between linear and asymptotic regimes (Stanley et al., 2017).

To model behavioral performance, two statistical approaches were employed:

1. Performance (fraction correct performance for each retrieval epoch) was entered into a mixed‐model analysis of variance (ANOVA) with group (HC vs. SCZ) as the independent variable, and memory block/time (1–8) as the within‐subjects (dependent) variable.
2. Fraction correct performance in each participant was modeled using the nonlinear least‐square fitting Gompertz function, which ideally characterizes negatively accelerated learning, represented in Equation (1):
  (1) $$\text{Fraction correct}=a\times e^{\left(-e^{\left(b-c\times \text{time}\right)}\right)}$$
  where *a* represents the asymptote (considered to reveal learning capacity), *b* represents the learning rate time constant, and *c* represents the inflection point (time at which the performance transitions from linear to asymptotic). Modeling was conducted using the *lsqnonlin* function in MATLAB (MathWorks, Inc.).

### 2.5 Time series and dFC analysis

**FIGURE 1:**
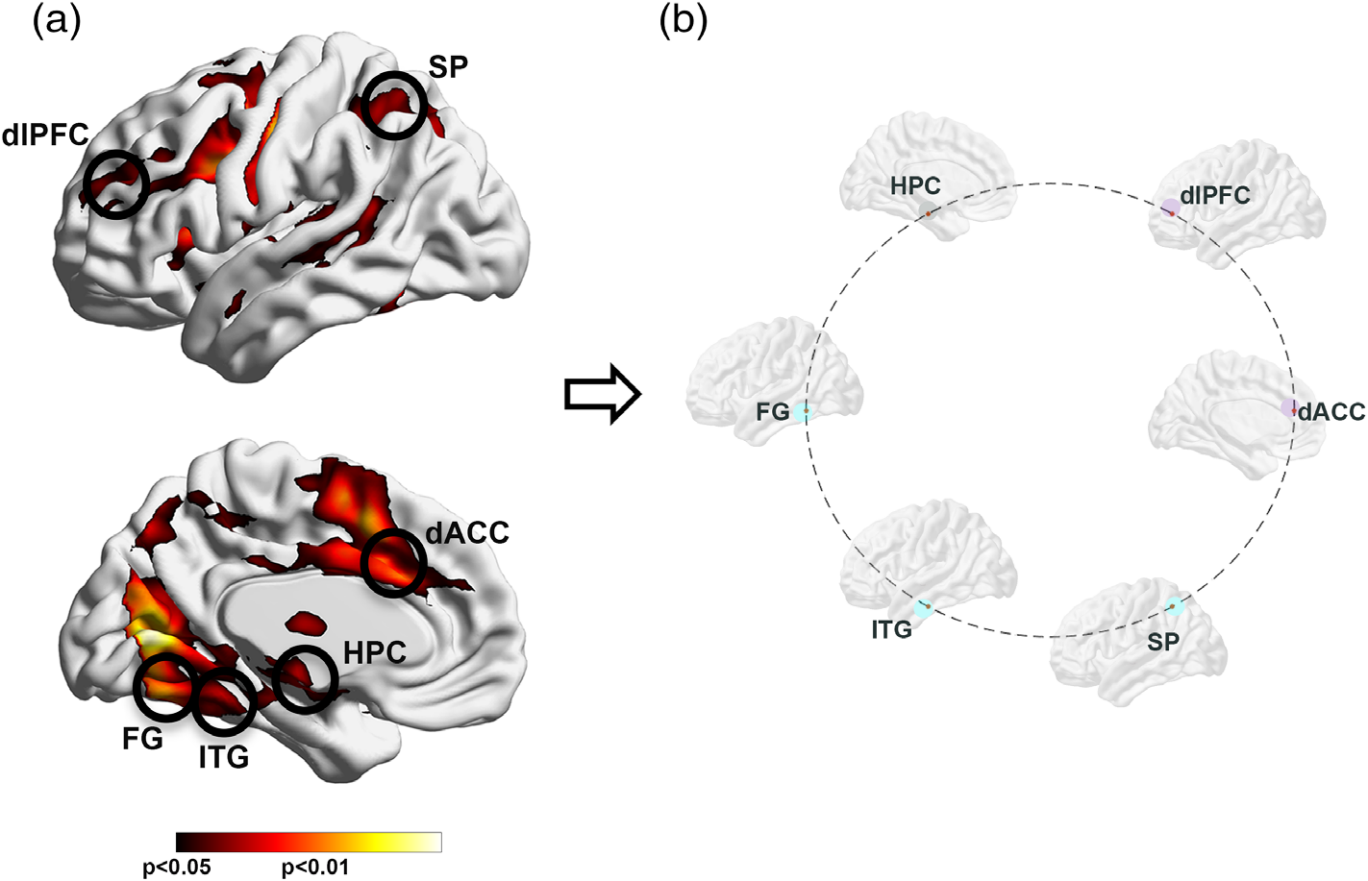
(a) The results of a conjunction analysis (SCZ ∩ HC) are projected to bilateral lateral and medial cortical surfaces. The significance peaks (insets) constitute a common substrate of *activation* across groups and conditions. These were harvested for subsequent dFC analyses, to avoid connectivity estimates from being confounded by activation differences, and to base dFC estimates on statistically filtered fMRI data. The harvested peaks represented the dorsolateral prefrontal cortex (dlPFC), the dorsal anterior cingulate (dACC), the hippocampus (HPC), the superior parietal cortex (SPC), the fusiform gyrus (FG), and the inferior temporal gyrus (ITG). (b) The schematic connectomic ring provides the framework for subsequent depiction of dFC results (Figures 3, 4, 5). The nodes are color coded by functional clusters; frontal/executive function (dlPFC, dACC; light purple), medial temporal lobe (HPC; gray), and unimodal function (FG, ITG, SP; teal). dFC, directional functional connectivity; HC, healthy controls; SCZ, schizophrenia

Coactivated nodes were identified using a conjunction analyses (HC ∩ SCZ) (Nichols, Brett, Andersson, Wager, & Poline, 2005) to identify a common functionally derived network across groups and epochs (ensuring that subsequent differences in dFC were not confounded by activation‐based differences) (Figure 1). Coactivated clusters were identified based on cluster‐level thresholding (*p* < .05, cluster level) (Ward, 2000) and centroids (radius = 5 mm) were established at the resultant significance peaks. Time series across participants (*n* = 55) from nodes in this functional network were forwarded for dFC analyses.

dFC was investigated within the MVAR statistical framework (Bressler & Seth, 2011; Diwadkar, Asemi, et al., 2017) (implemented in MATLAB) for using time series data from pairs of nodes (A, B), to estimate the strength of the directional effects between them (A ➔ B, B ➔ A).

Given two time series X and Y (representing dynamic state changes in nodes *j* and *i*), with *n* time points in each, the relationship between X and Y across all *n*, can be represented in the form of an MVAR model with the general representation:

(2) $$Z_{t}=\sum_{k=1}^{p}B_{k}Z_{t-k}+E_{t}$$

Here, *Z*_*t*_ is the dependent variable in vector form, representing the BOLD data values at arbitrary time *t* of all voxels in X and Y; *Z*_*t‐k*_ represents the values of the *Z* vector at and arbitrary earlier time point *t‐k*; lag *k* ranges from 1 to *p*, the model order; *B*_*k*_ is the corresponding coefficient matrix at lag *k*; and *E*_*t*_ is the residual vector.

The product term in Equation (2), *B*_k_ *Z*_*t‐k*_, is expanded into a matrix where each element of the *Z*_*t‐k*_th vector is a predictor, and each element (*b* ^*k*^_*ij*_) of the *B*_*k*_ matrix is a coefficient representing the degree of prediction of the *i*th element of *Z*_*t*_ by the *j*th predictor. If a value of *b* ^*k*^_*ij*_ significantly differs from zero, then significant “causality” is said to exist from node *j* to node *i*. The magnitude of the strength of the effect is represented in the model coefficient *b* (Morris et al., 2018; Tang et al., 2012),represents the degree of the causal relationship between the time series of nodal pairs, and is equivalent to GC (Granger, 1980). The significance of the effect can be assessed by the magnitude of the *t* statistic used to measure the difference of the *b* value from zero. Here, the MVAR model order (i.e., the number of previous time points in the model used to estimate a current time point), was one (Tang et al., 2012), consistent with our objectives, and with known limits of the temporal resolution of the fMRI signal in estimating network interactions (Logothetis, 2008). The method employed is made available online (https://github.com/WSUBRAINS/fMRI_MVAR_ANALYSIS).

To harness the dynamics of how dFC (and differences; HC ≠ SCZ) evolved over the course of the study, analyses were organized by phases of learning. This division separated the first four epochs of the task (linear increases in learning proficiency, henceforth “Early” learning) from the last four epochs of the task (when learning proficiency reached approximate asymptomatic performance, henceforth “Late” learning).

For each participant, MVAR coefficients were estimated for each of four conditions from a factorial combination of Epoch (Encoding vs. Retrieval) and Time (Early vs. Late), and for each direction. The resultant adjacency matrix for each participant in each condition consisted of 30 coefficients (6 nodes; 30 pairs, including both directions: A → B & B → A, and excluding on‐diagonal elements) providing a detailed picture of how directionality in network interactions during each phase of the task (Encoding vs. Retrieval) was dys‐modulated during the Early and the Late stages of learning.

MVAR coefficients were submitted for analyses of intergroup differences (HC ≠ SCZ, *q*_FDR_ < 0.05) (Benjamini & Hochberg, 1995). This comprehensive analytic framework provided estimates of time affected intergroup differences in dFC in each network pair (A, B) and direction (A ➔ B, B ➔ A), for each epoch type.

## 3 RESULTS

**FIGURE 2:**
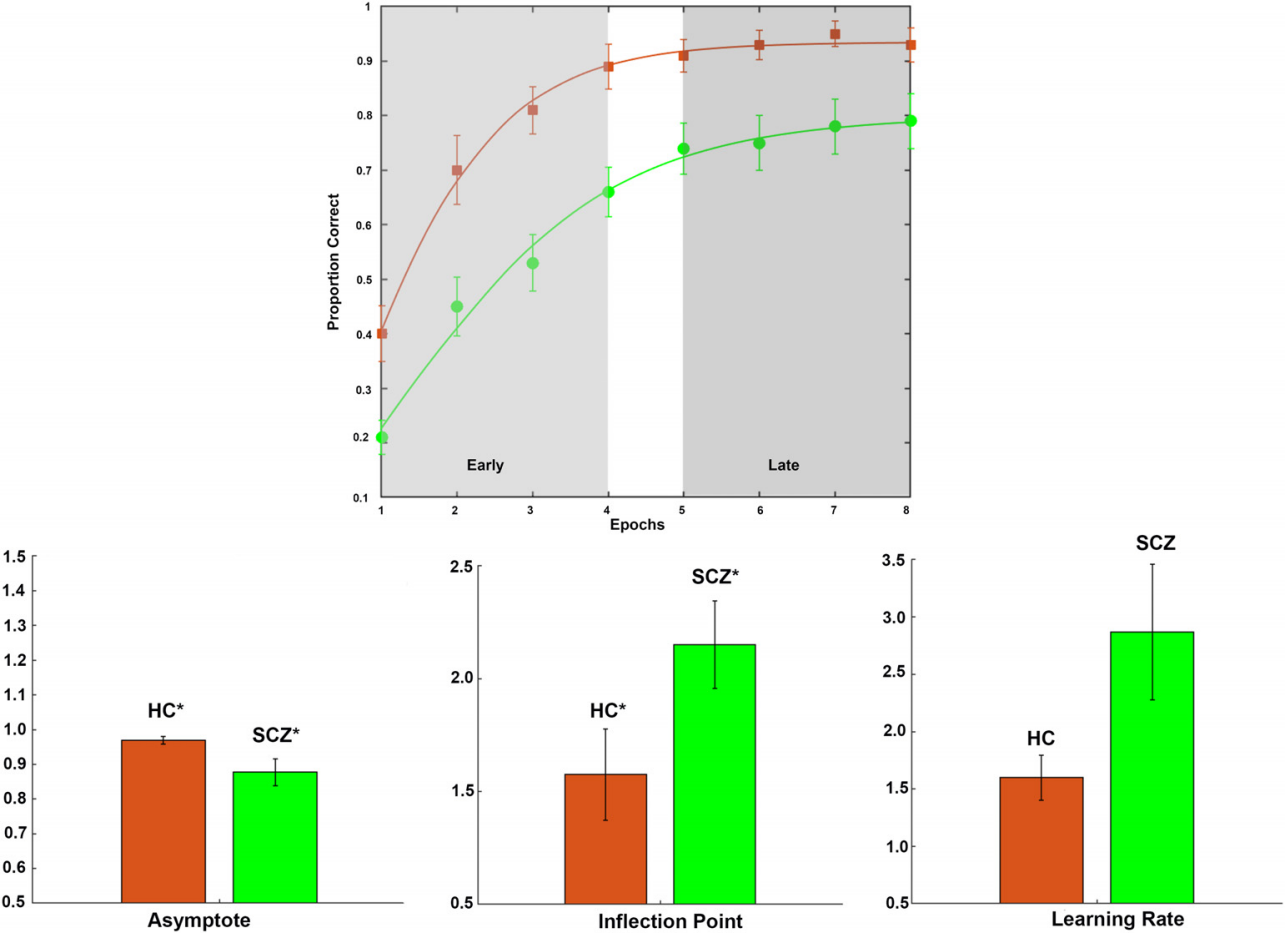
(a) The points represent recall performance at each of the eight retrieval epochs for healthy controls (HC, orange) and schizophrenia (SCZ, green) patients (error bars are ±*SEM*). The overlaid curves represent the Gompertz function fit to the average performance for each group. As evident from the shaded windows, the Early and Late phases of learning are characterized, respectively, by linear and asymptotic performance regimes. Subsequent figures represent the mean parameter estimates from fitting Gompertz functions to data from each individual participant. The data are presented for (b) asymptote, (c) learning rate time constant, and (d) inflection point (error bars are ±*SEM*). As seen, on average, SCZ patients reached lower asymptotic proficiency than HC (*p* < .05), and transitioned from linear to asymptotic learning later, (*p* < .05). The increase in the learning rate time constant was not statistically significant (*p* > 0.05) but is indicative of slower learning rates in SCZ patients

**FIGURE 3:**
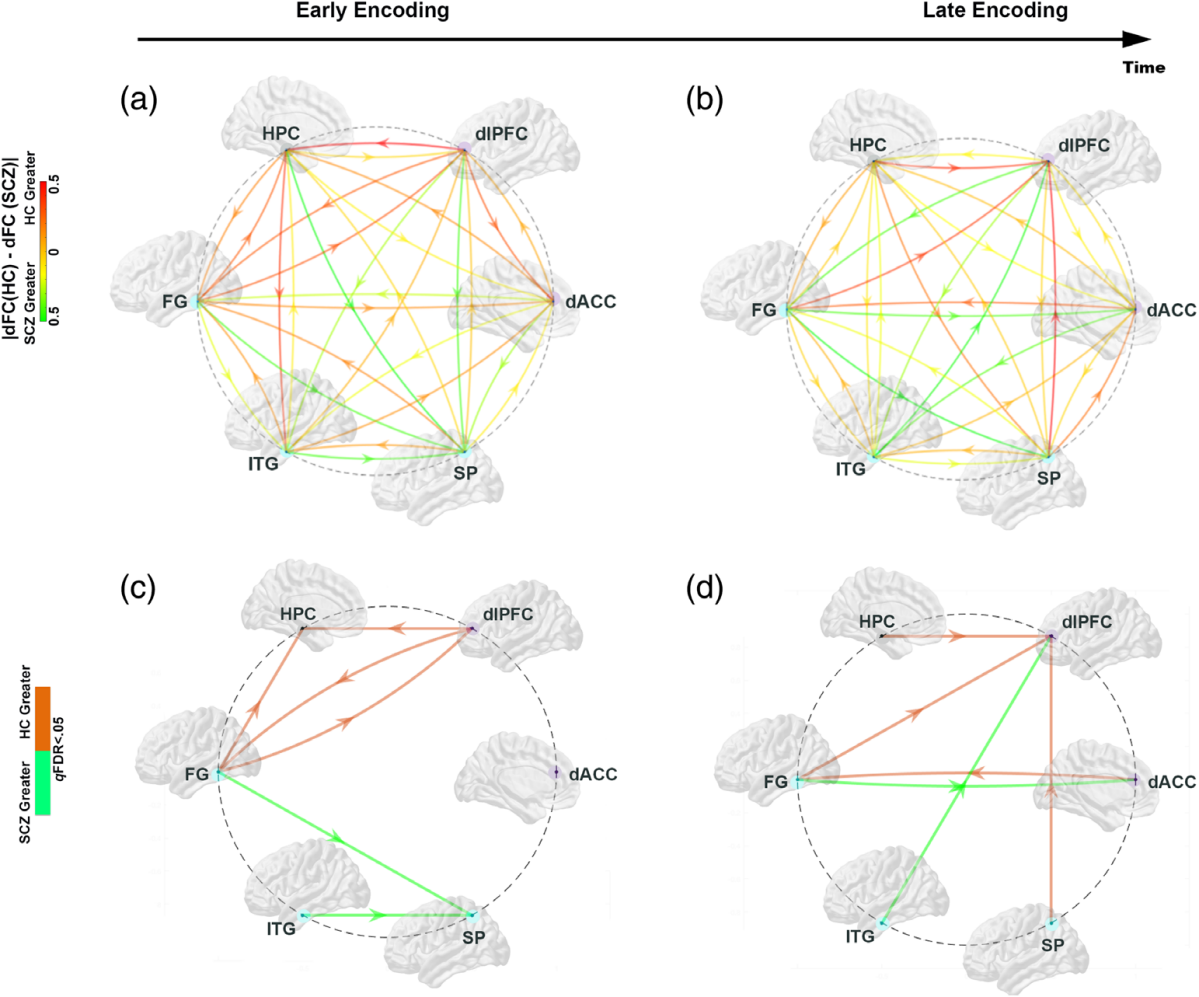
Using the connectomic ring (see Figure 1), for each of the pairwise subnetworks, we depict absolute *relative* differences (|dFC(HC)–dFC(SCZ)|) in the dFC values between groups (top row), and the significant differences (dFC(HC) ≠ dFC(SCZ); *p*_FDR_ < .05) between groups (bottom row). (a) Early Encoding: schizophrenia (SCZ) are characterized by relatively reduced dFC (warm/orange arrows) in the frontal (dlPFC)—hippocampal—FG network, with relatively increased dFC (cool/green arrows) from the FG and ITG to the SP. These effects are confirmed in the significance dysconnectome in (c). (b) Late Encoding: During Late Encoding, relative reductions in dFC in SCZ patients shifted to directional connections into the dlPFC from the SPC, FG and SP, and from the dACC to the FG (confirmed in the significance dysconnectome in (d). By comparison, in patients, relative increases in dFC were observed into frontal regions (dlPFC and dACC) from the ITG and FG, respectively

**FIGURE 4:**
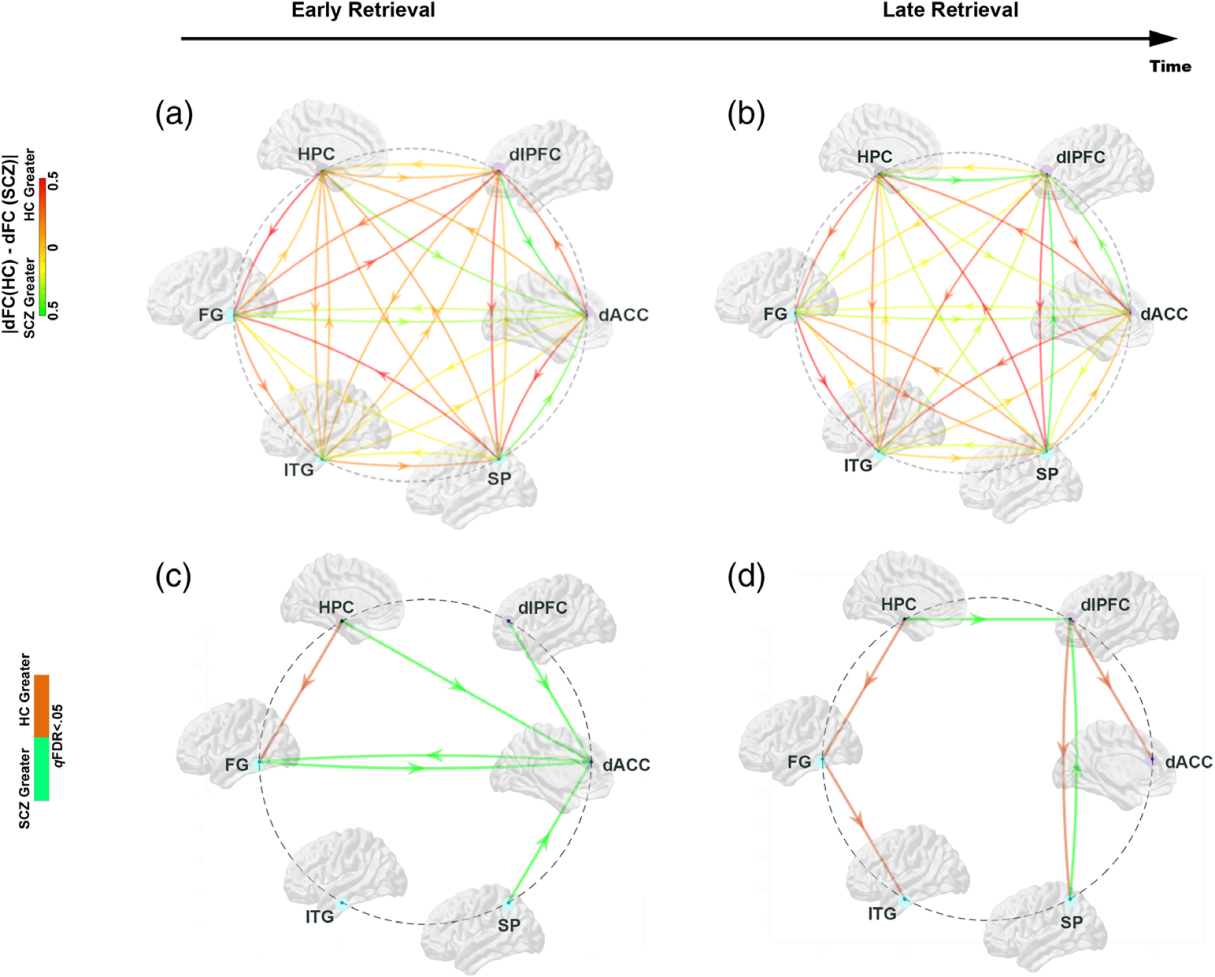
Absolute *relative* differences (|dFC(HC)–dFC(SCZ)|) in the dFC values between groups (top row), and the significant differences (dFC(HC) ≠ dFC(SCZ); *p*_FDR_ < .05) between groups (bottom row) are depicted during Retrieval. (a) Early Retrieval: schizophrenia (SCZ) are characterized by relatively increase dFC (cool/green arrows) into the dACC from multiple sources (dlPFC, HPC, FG, SP), and from the dACC to the FG. The only pathway with decreased dFC was the HPC ➔ FG. These effects are confirmed in the significance dysconnectome (c). (b) Late Retrieval: During Late Retrieval, relative increases in dFC in SCZ patients shifted to pathways to the dlPFC (from the HPC and the SP). Decreased dFC was observed on the HPC ➔ FG, FG ➔ ITG, dlPFC ➔ SP and dlPFC ➔ dACC pathways

**FIGURE 5:**
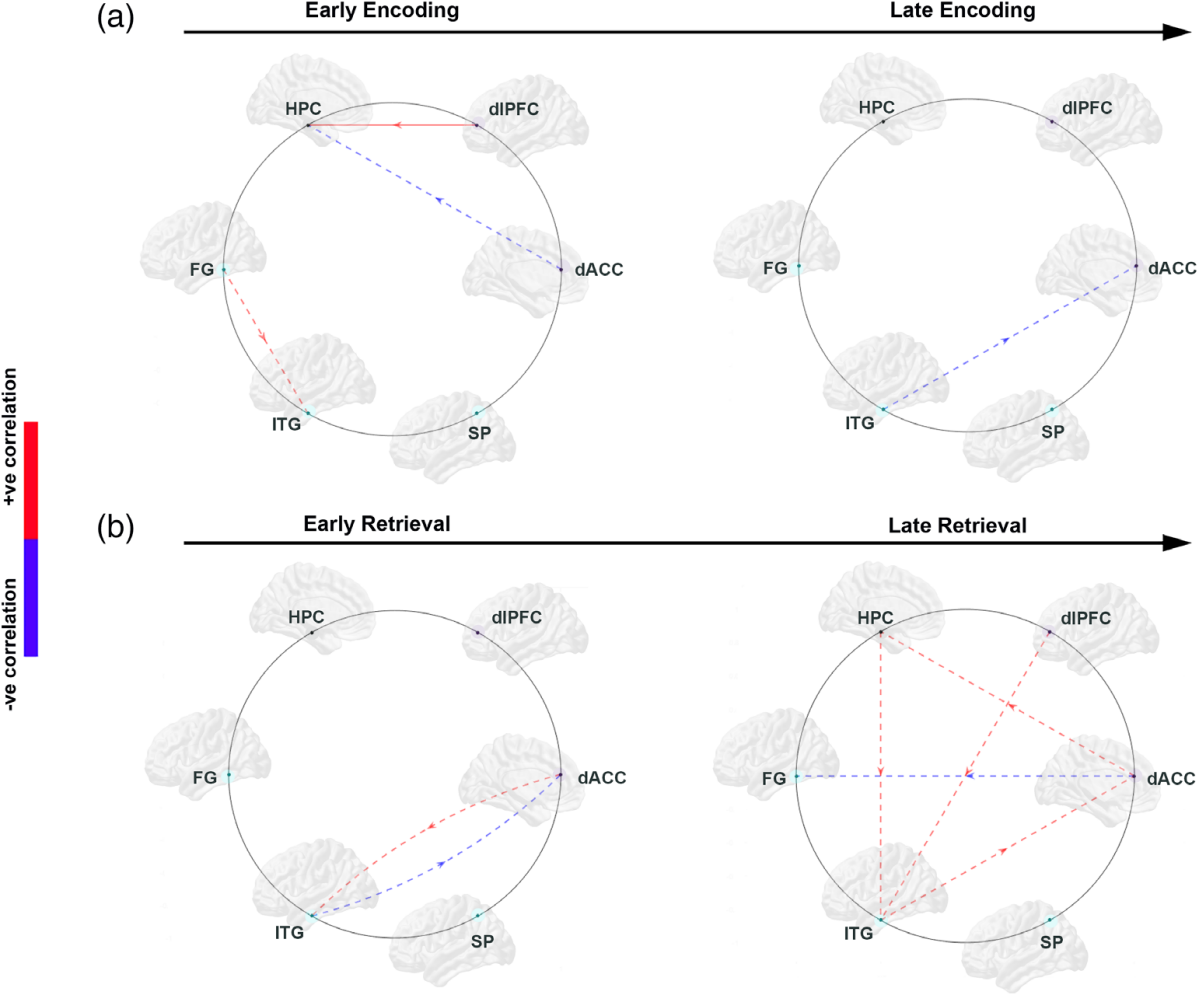
We investigated if in schizophrenia (SCZ) patients, medication dosage (see Methods) were positively (red) or negatively (blue) correlated with directional functional connectivity (dFC) parameters for each of the two learning phases associated with (a) Encoding and (b) Retrieval. These analyses identified an admixture of correlations across conditions and phases (identified in dashed lines: red—significant positive correlations; blue—significant negative correlations). However, the majority of effects were on pathways *not* implicated in patient‐control differences (Figures 3 and 4). A notable exception was a significant positive correlation on the dlPFC ➔ HPC during the early phase of encoding (denoted by a solid red line). The import of these effects is visited in Section 4

The results are organized as follows: (a) First (Figure 2), we present behavioral effects, with observed data, and patient‐control differences in learning parameter estimates; (b) Next (Figure 3), we present relative differences in the magnitude of dFC estimates (|dFC(HC)–dFC(SCZ)| for *Encoding* epochs (Figure 3a), represented as *weighted* edges in the underlying connectomic ring (carried forward from Figure 1b). From the dFC data, we derived significant differences between groups, represented as *binary* edges on the connectomic ring (Figure 3b). (c) Next (Figure 4), we present differences in dFC estimates (|dFC(HC)–dFC(SCZ)| and significant intergroup effects for the *Retrieval* epochs (Figure 4). (d) Finally (Figure 5), we report the results of exploratory analyses investigating the medication dosage effects on SCZ patients' behavioral performance as well as dFC estimates.

### 3.1 Behavioral results

The mixed‐model ANOVA resulted in a significant main effect of time (*F*_(1,41)_ = 133.82, *p* < .001, *MSe =* 0.070) with a large effect size (partial *η* ^2^ = .77), evidence that behavioral performance robustly improved (regardless of group). A significant main effect of group (*F*_(1,41)_ = 13.05, *p* < .01, *MSe =* 0.21) with a moderate effect size (partial *η* ^2^ = .24) was observed, indicating impaired overall memory performance in SCZ compared to HC. Figure 2a shows the average performance data for HC (blue) and SCZ (red). The curves represent Gompertz functions fit to the *average* HC and SCZ data. The shaded portions of the learning functions clearly delineate differences between Early (Linear) and Late (Asymptotic) learning, which motivated understanding of the network correlates of learning dynamics.

The bar graphs depict the mean estimates of performance parameters ([b] asymptote, [c] learning rate time constant, and [d] inflection point) for healthy controls compared to SCZ (±*SEM*) derived from Gompertz functions fit to *individual* participants' data. As shown, on average, SCZ reached lower asymptotic proficiency than healthy controls (*p* < .05; Figure 2b) with a moderate effect size (Cohen's *d* = .63), evidence for a reduction in learning capacity (Diwadkar et al., 2008). Patients transitioned from linear to asymptotic learning later, (*p* < .05; Figure 2c) with a large effect size (Cohen's *d* = 1.04). The increase in learning rate time constant (Figure 2d) was not statistically significant (*p* > .05) but is suggestive of slower learning rates in SCZ.

### 3.2 Exploratory analysis of age and FSIQ effects

We also assessed the statistical effects of FSIQ (Wechsler, 2011) and age on multiple dependent variables including both (a) behavioral metrics and (b) MVAR coefficients. Age and FSIQ data were submitted to regression models to examine their statistical effects on, (a) fraction correct data (that is the average ratio of correctly recalled items to total items across Early and Late epochs), (b) the modeled performance parameters for learning rate and inflection point, and (c) MVAR coefficients for all subnetwork pairs and directions. For the MVAR coefficients, the analyses were conducted for coefficients associated with *each* task condition (Encoding, Retrieval) and *each* Phase (Early, Late) (i.e., 30 directional interactions between the six‐node network). Significant correlations were identified using statistical thresholds (*q*_FDR_ < 0.05). In these exploratory analyses, age did not exert any significant effect on any of the behavioral performance parameters. However, consistent with previous studies (Mohn, Sundet, & Rund, 2014), FSIQ predicted behavioral metrics. An increase in FSIQ was predictive of increased learning proficiency during both Early (*r* = .52) and Late periods (*r* = .31). FSIQ did not predict any of the dFC parameter values. With the absence of behavioral proficiency, it appears that neither participant Age nor FSIQ were predictive of the observed connectivity measures.

### 3.3 Directional functional connectivity

#### 3.3.1 Memory encoding

In each connectomic ring (Figure 3a,b), we depict relative differences in dFC values during each of the Encoding phases. In the color scheme (maintained going forward), warm colors indicate reduced dFC in SCZ (i.e., an increase in HC compared to SCZ), whereas cool colors indicated the converse.

As indicated by the relative dFC effects, during Early Encoding, SCZ were characterized by reduced dFC within a network of regions that included the dlPFC, the Hippocampus and the FG. By comparison, SCZ appeared to be characterized by increased dFC into the SP. Significance rings (Figure 3c) confirmed these effects: Significantly reduced dFC was observed in SCZ for: dlPFC ➔ hippocampus, FG ➔ hippocampus, and bidirectionally between the dlPFC and the FG. By comparison, SCZ were characterized by increased dFC for FG ➔ SP and ITG ➔ SP.

During Late Encoding, the dFC differences became more evident in pathways leading to and from frontal regions, specifically the dlPFC and the dACC (Figure 3d). As seen, in SCZ, dFC was reduced for: HPC ➔ dlPFC, FG ➔ dlPFC, SP ➔ dlPFC, and dACC ➔ FG. In comparison, in SCZ there was increased dFC in the ITG ➔ dlPFC and FG ➔ dACC pathways. These effects were confirmed in the significance rings below.

#### 3.3.2 Memory retrieval

As shown in Figure 4c, during Early Retrieval, contrasting patterns of dFC were observed. SCZ were characterized by decreased dFC on the HPD ➔ FG pathway. By comparison, statistically significant increases in dFC in SCZ were observed on: dlPFC ➔ dACC, HPC ➔ dACC, SP ➔ dACC, FG ➕➔ dACC (bidirectionally).

During Late Retrieval, SCZ were characterized by significantly reduced dFC (Figure 4d) on: dlPFC ➔ SP, dlPFC ➔ dACC, HPC ➔ FG, FG ➔ ITG, but increased dFC for the pathways leading to the dlPFC from the HPC and SP (HPC ➔ dlPFC; SP ➔ dlPFC).

### 3.4 Exploratory analysis of medication effects in the SCZ group

Because exposure to psychotropic medication can exert effects on activation and connectivity metrics (Abbott et al., 2011; Abbott, Jaramillo, Wilcox, & Hamilton, 2013), we explored potential effects of medication dosage on learning performance and MVAR coefficients. To achieve this, we quantified dosage‐related effects on both (a) behavioral performance, and (b) MVAR coefficients for each of the 30 directional interactions between the six‐node network. Analyses were conducted for each of the task conditions (Encoding, Retrieval) and Phases (Early, Late). Significant correlations were thresholded (*q*_FDR_ < 0.05).

Dosage was quantified based on the ratio of the prescribed daily dose (PDD) and defined daily dose (DDD) (Nose & Barbui, 2008). The PDD/DDD ratios for each SCZ patient were submitted to separate regression models against: (a) fraction correct data: average ratio of correctly recalled items to total items across Early and Late epochs, (b) performance parameters: learning rate and inflection point, and (c) MVAR coefficients for all subnetwork pairs and directions.

Whereas psychotropic dosage had no significant effect on any of the parameters for behavioral performance (*p*s: .23–.75), significant effects of dosage were observed on a subset of MVAR coefficients (Figure 5). Blue colors indicate a significant *negative* correlation between MVAR coefficients and psychotic dosage, while red colors indicate a significant *positive* correlation between MVAR coefficients and psychotic dosage. The effects are distinguished based on whether the pathway was significantly different in the intergroup analyses (HC ≠ SCZ, Figures 3 and 4, solid lines), or not (dotted lines).

As seen, the set of significant pathways in which antipsychotic dosage in patients predicted MVAR coefficients largely nonoverlapping with the set of significant intergroup (HC ≠ SCZ) differences (Figures 2 and 3). A notable exception was the dlPFC ➔ HPC pathway (*r* = .29). Thus, within patients, medication predicted connectivity changes on pathways that (but for the single noted exception) were not different between patients and controls. The import of these effects is visited in Section 4.

## 4 DISCUSSION

We explored patient—control differences in dFC (estimated using MVAR models) induced by associative learning with negatively accelerated learning dynamics (Figure 2). Our salient results were as follows:

1. During Early Encoding (Figure 3a,c), SCZ were characterized by reduced dFC within a frontal–hippocampal–FG network, though during Late Encoding (Figure 3b,d) reduced dFC was associated with pathways *toward* the dlPFC.
2. During Early Retrieval (Figure 4a,c), SCZ were characterized by increased dFC in pathways mainly associated with the dACC, though during Late Retrieval (Figure 4b,d), patients were characterized by increased dFC in pathways directed *toward* the dlPFC, but decreased dFC in the pathways *from* the dlPFC.
3. These effects were largely unrelated to FSIQ, age, and medication (Figure 5), though neuroleptic dosage exerted some effects on dFC.

Recent SCZ studies have used Granger causality to investigate network interactions associated with resting‐state fMRI signals (Huang et al., 2018; Iwabuchi & Palaniyappan, 2017), working memory (Pu et al., 2016), and during episodic memory retrieval (Hutcheson et al., 2015). However, our results are singular in depicting dysfunctional directionality induced during associative memory encoding, retrieval and their temporal dynamics. The results highlight the salience of frontal–hippocampal interactions during early memory acquisition (Raynal, Schnider, & Manuel, 2019), and of the importance of hippocampal–neocortical interactions in the initial stages of (the eventually prolonged process of) memory consolidation (Haist, Bowden Gore, & Mao, 2001). Moreover, they provide a directional framework to underpin hippocampal functional deficits in SCZ (Ragland et al., 2017). These themes, and potential mechanisms discovered by our analyses are visited in the remainder of Section 4.

### 4.1 Memory dynamics and dysfunctional directional interactions during encoding

Memory consolidation emerges through dynamics involving the medial temporal lobe and the neocortex (Wiltgen & Tanaka, 2013). Although consolidation generally encompasses encoding *and* retrieval, each subprocess is expected to induce distinct effects during learning (Simons & Spiers, 2003). The in‐task evolution of patient‐control differences during Encoding (Figure 3) is revealing for reflecting the time dependence of circuit deficits in SCZ (Bontempi, Laurent‐Demir, Destrade, & Jaffard, 1999; Mishkin, Vargha‐Khadem, & Gadian, 1998). Early encoding induced reductions in interactions for dlPFC ➔ HPC, and bidirectional interactions between dlPFC and the FG. The former effects can be related to (a) recent studies in mice showing that (optogenetic) inhibition of excitatory medial prefrontal cortical neurons inhibits activation of the entorhinal–hippocampal circuit, in turn inhibiting long term memory formation (Bero et al., 2014), and (b) fMRI studies at the macroscopic scale that have reaffirmed the role of disrupted cognitive control during episodic memory formation (Ragland et al., 2015) and learning in SCZ (Woodcock et al., 2016).

Thus, loss of directional interactions of dlPFC ➔ HPC (and the FG) during early memory encoding suggests a disruption of “top‐down” mechanisms of frontal control material at early stages of memory formation (Crane & Milner, 2005). Loss of bidirectional causality between the dlPFC and FG pathway confirms previously documented deficits in ventral‐stream processing (Sehatpour et al., 2010), that also reflect structural and “connectivity” deficits of the FG (Abrol, Rashid, Rachakonda, Damaraju, & Calhoun, 2017).

Activation‐based meta‐analyses suggest that SCZ patients are characterized by “overactivation” in network nodes deemed peripheral rather than central in the connectome (Crossley et al., 2016). These studies moderately inform the interpretation of our connectivity analyses, because increases in connectivity for FG ➔ SP and ITG ➔ SP pathways suggest that the early phase of encoding associations is associated with relatively inefficient transactions between ventral (FG and ITG) *and* dorsal (SP) visual stream nodes which are associated with the processing of object identity and spatial location, respectively (Mishkin, Ungerleider, & Macko, 1983).

Later stages of encoding were characterized by reduced directional interactions into the dlPFC from the HPC, FG, and SP. Thus, during asymptotic memory performance, there is a reduced “flow” of information into the dlPFC from ventral and dorsal stream areas, and from the hippocampus. These effects emphasize the central role of the dlPFC (and hippocampus) during later stages of memory consolidation (Zhan, Guo, Chen, & Yang, 2018) when hippocampal traces are redistributed into the neocortex (Remondes & Schuman, 2004). Moreover, patients were also characterized by reduced directional interactions from the dACC to the FG, confirming that mechanisms of “memory control” that are part of the repertoire of the anterior cingulate (Bubb, Metzler‐Baddeley, & Aggleton, 2018), are impacted during late phases of encoding. Significantly increased directional interactions were also observed for the ITG ➔ dlPFC and the FG ➔ dACC. The pathways and targets are unique, but both effects are in the “bottom up” direction, suggesting inefficient unidirectional information flow in the late stages of learning in SCZ.

### 4.2 Memory dynamics and dysfunctional directional interactions during retrieval

Functional connectivity analyses link the retrieval of memories to network‐wide interactions between the hippocampus, dlPFC, and the dorsal anterior cingulate (Geib, Stanley, Dennis, Woldorff, & Cabeza, 2017), independent of the *content* of memoranda, and other content specific regions (Rugg & Vilberg, 2013). In this context, patterns of hypo‐ and hyper‐directionality, and how these patterns relate to dysfunctional dynamics in SCZ are revealing. Explicit memory retrieval is resource intensive and demanding (Reas & Brewer, 2013), and is unsurprisingly associated with performance declines in conditions such as aging (Clark, Hazeltine, Freedberg, & Voss, 2018; Diwadkar et al., 2016). As seen, patients were characterized by increased directional interactions on multiple pathways converging *on* the dACC (from the hippocampus, dlPFC, FG, and SP), and *from* the dACC to the FG. These effects during embryonic stages of the task (when the relative immaturity of memory traces results in demanding retrieval), complement the *hypo‐*directional effects observed in patients during early encoding. Impaired integrity of directional network interactions during the early phases of Encoding appear *to have to be compensated* for by hyper‐directional interactions during the corresponding early phases of Retrieval. Clearly, the dACC plays a central role in the context of memory control (Diwadkar, Re, et al., 2017; Woodcock et al., 2015) (see Rajasethupathy et al., 2015 for evidence of an anatomical basis for top‐down, i.e., cingulate ➔ hippocampus mediation).

In the final phase of Retrieval, patients were marked by *reduced* bidirectional interactions between the dlPFC and the SP and the dlPFC and the dACC,reduced directional interactions for the HPC ➔ FG, and FG ➔ ITG, but *increased* directional interactions converging into the dlPFC from the SP and the HPC. The hypo‐directionality from the dLPFC, may reflect a loss of effective cueing of retrieval from the frontal lobe, consistent with a hypothesized role for the frontal cortex in memory retrieval (Simons & Spiers, 2003), and the effects of frontal–hippocampal asynchrony during working memory in SCZ (Kupferschmidt & Gordon, 2018; Schneider et al., 2017).

### 4.3 Medication effects

Antipsychotic dosage exerted an admixture of effects on dFC, but on pathways orthogonal to patient‐control differences. During Encoding, negative correlations between dosage and dFC estimates were observed for dACC ➔ HPC (Early) and ITG ➔ dACC (Late), but positive correlations for FG ➔ ITG (Early) and dlPFC ➔ HPC (Early). Only the last pathway was represented in the patient‐control dysconnectome. It is tempting to overinterpret this final effect given that Hutcheson et al. have shown that a week of antipsychotic treatment (risperidone) increases bidirectional effects (also estimated using GC) during the retrieval of episodic memories (Hutcheson et al., 2015). However, our results are a naturalistic finding (dosing was uncontrolled), and in the context of a task with demands different from one‐shot episodic memory and retrieval. Nevertheless, that two independent studies (using substantively different paradigms) should reveal medication‐related effects on a frontal ‐ hippocampal pathway motivates further inquiry on the general nature of this effect.

Medication generally predicted significant increases in estimated connectivity during retrieval (Early and Late), notably emanating from the dACC (Early and Late) and the dlPFC (Late). These results confirm the sporadic effects that psychotropic medication exerts on general connectivity measures in fMRI data collected in SCZ patients (Cadena et al., 2019; Lottman et al., 2017).

### 4.4 What do these revelations contribute to the state of the dysconnection hypothesis?

The dysconnection hypothesis attempts to link the symptoms of SCZ, with the brain's molecular and neuronal pathophysiology (Friston et al., 2016), a rational approach consistent with modern scientific approaches to the study of multiple branches of medicine. The explicit idea is that psychosis is best understood as a *systemic* rather than a local dysfunction, that results from aberrant neuromodulation of synaptic efficacy which in turn mediates context‐sensitive influences on “connectivity.” It proposes that a key aspect of the illness' pathophysiology lies in the interactions between NMDA receptor function and modulatory neurotransmitter systems (Stephan, Friston, & Frith, 2009). The dysconnection hypothesis, or more specifically the syndrome, cannot be captured in any single study; after all, the brain is *both* a “statistical” organ (Dayan, Hinton, Neal, & Zemel, 1995) and a “contextual” organ (Park & Friston, 2013). As the former, it has evolved to actively model the environment while simultaneously evaluating sensory evidence against a set of internal formal representations, an idea that found its earliest expression in linguistics (Chomsky, 1957). As the latter, its functional expressions are only loosely constrained by its underlying structure (Batista‐Garcia‐Ramo & Fernandez‐Verdecia, 2018; Pernice, Staude, Cardanobile, & Rotter, 2011). Brain function and dysfunction are inherently dynamic constructs, just as psychosis is itself a dynamic expression of an underlying trait that emerges from a cluster of disease properties (Kendler, Zachar, & Craver, 2011). Indeed, our results imply that even within the context of a time‐limited experimental manipulation, directional network interactions in SCZ change in meaningful ways. Thus, the dysconnection hypothesis must endeavor to reveal the how *task‐induced* effects evoke dysfunctional brain dynamics in SCZ. At its core, the “dysconnection syndrome” is not “a thing” but a set of emergent properties that are dynamic expressions of ingrained pathological processes in the brain.

### 4.5 Conclusions

We infer that in SCZ the early stages of memory formation are characterized by a loss of *directional* consistency between subnetworks crucial in processes of memory formation and consolidation (Rusu & Pennartz, 2019). During complementary periods of Early Retrieval, this loss appears to be “compensated” for by interactions from and to the dACC, a region the dysfunction of which is heavily implicated in SCZ (Bubb et al., 2018). Specific pathways (FG ➔ dlPFC, Encoding; HPC ➔ FG, Retrieval) showed reduced dFC across both phases, but learning dynamics induced largely nonoverlapping patterns of dysfunction during both Encoding and Retrieval.

MVAR models have been considered controversial for fMRI analyses (Smith et al., 2012). Challenges to interpretation include hemodynamic variation across regions, challenges of using temporal precedence in estimating causal interactions (Friston, Moran, & Seth, 2013), and limitations in the statistical model itself (Silverstein et al., 2016). However, extensive evidence based on experimental and simulated BOLD data (Deshpande & Hu, 2012; Deshpande, Sathian, & Hu, 2010; Duggento, Passamonti, Guerrisi, & Toschi, 2018; Rodrigues & Andrade, 2014) have affirmed the robustness of Granger causality in estimating directional relationships (or neuronal “causality”), particularly in task‐constrained data. Moreover, as has been recently shown, the recovered information is complementary in meaningful ways, to what is recovered with nondirectional models (Morris et al., 2018). Finally, MVAR models lie within a class of “weak” models of directional functional interactions between nodes in brain networks, and lack the power of approaches such as dynamic causal modeling (DCM) (Friston et al., 2019). DCM relies on a well‐validated neural mass model of fMRI time series data to target effective connectivity (and perturbation‐induced changes) in a system. Thus, MVAR models can provide useful insights into any system's dynamical behavior under different conditions albeit in a piecemeal (node‐to‐node) manner, but subsequent investigations can be underpinned by stronger “mechanistic” approaches like DCM, that permit assessment of changes within a finite system. This remains a central ambition of our ongoing work in this area.

Understanding “causality” in brain networks is a nontrivial challenge, the complexities of which are frequently not contemplated (Mannino & Bressler, 2015). However, the application of directed connectivity methods of which MVAR models are a class, should be an essential tool in the service of elucidating new vistas for the dysconnection syndrome that is SCZ (Friston et al., 2016).

## CONFLICT OF INTEREST

The authors declare no potential conflicts of interest.

## Data Availability

Data available upon request.
